# A comparison of two methods for expert elicitation in health technology assessments

**DOI:** 10.1186/s12874-016-0186-3

**Published:** 2016-07-26

**Authors:** Bogdan Grigore, Jaime Peters, Christopher Hyde, Ken Stein

**Affiliations:** Evidence Synthesis & Modelling for Health Improvement (ESMI), Institute for Health Research, University of Exeter Medical School, University of Exeter, Room 3.09.3, St Luke’s Campus, Heavitree Road, Exeter, EX1 2LU UK

**Keywords:** Expert elicitation, Probability distributions, Parameter uncertainty, Economic evaluation, Health technology assessment

## Abstract

**Background:**

When data needed to inform parameters in decision models are lacking, formal elicitation of expert judgement can be used to characterise parameter uncertainty. Although numerous methods for eliciting expert opinion as probability distributions exist, there is little research to suggest whether one method is more useful than any other method. This study had three objectives: (i) to obtain subjective probability distributions characterising parameter uncertainty in the context of a health technology assessment; (ii) to compare two elicitation methods by eliciting the same parameters in different ways; (iii) to collect subjective preferences of the experts for the different elicitation methods used.

**Methods:**

Twenty-seven clinical experts were invited to participate in an elicitation exercise to inform a published model-based cost-effectiveness analysis of alternative treatments for prostate cancer. Participants were individually asked to express their judgements as probability distributions using two different methods – the histogram and hybrid elicitation methods – presented in a random order.

Individual distributions were mathematically aggregated across experts with and without weighting. The resulting combined distributions were used in the probabilistic analysis of the decision model and mean incremental cost-effectiveness ratios and the expected values of perfect information (EVPI) were calculated for each method, and compared with the original cost-effectiveness analysis.

Scores on the ease of use of the two methods and the extent to which the probability distributions obtained from each method accurately reflected the expert’s opinion were also recorded.

**Results:**

Six experts completed the task. Mean ICERs from the probabilistic analysis ranged between £162,600–£175,500 per quality-adjusted life year (QALY) depending on the elicitation and weighting methods used. Compared to having no information, use of expert opinion decreased decision uncertainty: the EVPI value at the £30,000 per QALY threshold decreased by 74–86 % from the original cost-effectiveness analysis. Experts indicated that the histogram method was easier to use, but attributed a perception of more accuracy to the hybrid method.

**Conclusions:**

Inclusion of expert elicitation can decrease decision uncertainty. Here, choice of method did not affect the overall cost-effectiveness conclusions, but researchers intending to use expert elicitation need to be aware of the impact different methods could have.

**Electronic supplementary material:**

The online version of this article (doi:10.1186/s12874-016-0186-3) contains supplementary material, which is available to authorized users.

## Background

Decision models are frequently used to synthesise evidence on cost-effectiveness of health interventions in health technology assessment (HTA). The models can be used as a support tool by policy makers to optimise health care delivery. However, policy makers are often confronted with having to make decisions even when evidence is scarce or lacking; delaying the decision until more evidence is available carries a risk of utility loss, and is not always possible, due to legal constraints, or the fact that such evidence may never become available.

With the trend towards probabilistic decision-analytic models in HTA [1], there is a need that the data informing the model parameters is available as distributions.

In such cases, expert opinion can be used to characterise the different types of model uncertainty; this can also be used in value of information analyses to help identify future evidence needs (including the type of study design, sample size) for reducing the decision uncertainty [2]. However, the majority of available literature addresses methods for obtaining single values for quantities of interest, without associated uncertainty [3]. This is also reflected in the relatively few attempts to encode expert judgements as distributions – process we will refer to as expert elicitation – in HTA. A previous review [4] of reported use of elicitation in HTA found a limited number of reports and great variation in the approaches used. There was also limited information on how authors choose among available elicitation methods, although the review suggests that ease of use is an important criterion [4].

Expert elicitation is a complex task. Many factors have an impact on the elicited distributions, including those related to the expert (e.g. motivational bias), to the elicitation methods used or to the specific quantity investigated [5]. In practice, the choice of the elicitation approach usually depends on the constraints of the broader decision analysis process, which include availability of experts, experts’ familiarity with probabilistic judgement, time available and funding [6, 7].

The elicitation literature describes numerous methods to represent expert opinion as probability distributions [7–9], and comparative studies [10–13] show that different methods could lead to different results. However, objectively choosing one method over the others is challenging.

The lack of a clear approach to evaluating these methods [14] is a key reason why choosing methods is difficult. For instance, one of the widely accepted dimensions of elicitation is its ability to accurately capture expert’s belief [5]. This ability cannot be measured objectively, because only the expert can judge how accurately his or her belief has been recorded. Consequently, analysts have to settle for the feedback obtained from the experts themselves to assess if elicitation was “well done”.

Some qualities of the expert are critical for a successful elicitation. Apart from possessing substantive knowledge in their field, experts also need to be able to engage with the elicitation method proposed to them and provide consistent judgements [5]. At the same time, the identification of a clinician as an “expert” does not ensure that the expert also possesses normative expertise (the ability to provide numerical judgements for decision making processes). Even so, the estimates of a certain expert might be required even if the expert lacks normative goodness.

One characteristic of experts that has captured a lot of interest in the elicitation literature is their ability to make judgements that match empirical data; this characteristic is referred to as calibration [15]. A measure of how well experts are calibrated can be obtained by using seed variables – asking experts questions about quantities that are known to the analyst, but unlikely to be known by the expert. The assumption is that the expert performance on the seed variables predicts their ability to make judgements on the quantities of interest [15]. While a lot of experience has been gathered over time on using this model [16], selecting appropriate seeds is notoriously difficult, to the point that it can negate their usefulness [17, 18].

The process of elicitation in the current study was explored by observing how two different elicitation methods performed in terms of their appeal for the experts, to see if any differences between preferences or elicited distributions were observed. As this study was conducted in the context of an existing HTA, the impact of elicited distributions was also examined in an actual decision model.

We set out with the following objectives: (i) to obtain subjective probability distributions characterising parameter uncertainty in a HTA decision model of a treatment for advanced-hormone dependent prostate cancer; (ii) to compare two elicitation methods by eliciting the same parameters in different ways; (iii) to collect subjective preferences of the experts for the different elicitation methods used.

In the next section we describe the decision analysis context, the elicitation methods used and the design of the comparative study, including expert selection and the analysis of responses.

## Methods

To minimise bias, the elicitation literature recommends a number of good practice steps to take when conducting expert elicitation [9]. These measures were implemented in the current study and included: preparation of the expert for the session, formal framework of the elicitation session, obtaining feedback from the expert and offering the possibility of adjusting their response.

### Illustrative decision analysis

A published probabilistic decision model [19] that could benefit from being informed by subjective probability distributions, provided the real-world context. This was an Excel-based decision model to evaluate the cost-effectiveness of degarelix versus triptorelin for the treatment of advanced hormone-dependent prostate cancer from the perspective of the National Health System in the United Kingdom. It consisted of a decision tree to simulate a hypothetical cohort of patients aged 70 years for the first month of hormonal treatment, and a Markov model to capture the longer term patient-related outcomes and costs for a time horizon of 10 years. Results from the model were reported in terms of the cost per quality adjusted life-year (QALY) gained, and deterministic, probabilistic and one-way sensitivity analyses were undertaken. One key element that differentiated the effectiveness of the two interventions was that triptorelin therapy causes an initial flare in testosterone levels, which may lead to serious clinical symptoms [20]. The proportion of severe cases of spinal cord compression as a result of the testosterone flare was a relevant parameter that could not be informed by available literature. In the original probabilistic cost-effectiveness analysis, this parameter was assumed to have a uniform distribution between 0 and 1, with a mean of 0.5. One-way sensitivity analyses indicated that the model was sensitive to this parameter, therefore obtaining more information, via expert opinion, could help reduce uncertainty in the analysis results.

### Elicitation methods

The two elicitation methods chosen for this study were identified as having previously been used in expert elicitation in the HTA context [4]: the histogram method and the hybrid method. Both methods are direct elicitation methods, meaning that experts provide a number of points on the probability density function (PDF), rather than provide parameters of the distribution [5].

The histogram method is a discrete form of the PDF, that allows quantities of interest to be elicited graphically [9]. The expert is presented with a frequency chart on which they are asked to place a number of crosses (alternatively called chips or tokens) to represent their uncertainty about the elicited quantity. Placing all the crosses in one column would represent complete certainty, while placing all the crosses on the bottom row would represent complete uncertainty. The histogram technique has been referred to by different names, including “chips and bins” [14] and the Trial Roulette method [21].

The hybrid method consists of eliciting the lowest (L), highest (H) and most likely value (M) of the quantity of interest from the expert. Intervals are automatically built using a formula to divide the distance between each extreme (L and H) and M into equal parts. Experts are then asked to enter the probability that their estimated value lays within each interval. Based on the work by Leal et al. [22], who found better compliance with the four interval version than the six interval version, as well as of other authors [23], the four interval version was used here.

### Study design

The study had a cross-over design, where each expert was asked to provide answers using the two different elicitation methods during a face-to-face interview.

The order in which the methods were applied was randomised. The same elicitation questions were asked using both methods:

1. *seed question* (question for which the answer is known to the investigator but not to the expert). “Consider the entire population of patients with metastatic prostate cancer starting treatment with a luteinising hormone-releasing hormone analogue (LHRHa). What proportion of these patients experience clinically significant complications as a result of testosterone ‘flare’?”
2. *main question:* “Consider the entire population of patients with metastatic prostate cancer experiencing spinal cord compression (SCC) as a result of testosterone flare on starting treatment with a luteinising hormone-releasing hormone analogue (LHRHa). What proportion of them would you expect to experience paraplegia?” The answer to this elicitation question would inform the parameter of interest in the HTA model.

More detail on the questionnaire is presented in the Additional file 1.

After completing each of the elicitation questions, regardless of the elicitation method, experts were shown a histogram summarising their elicited estimates of the quantity and associated uncertainty, and were given the possibility to adjust their answers.

In order to limit the impact of anchoring (experts fixing on values from previous questions [5]), a “cooling off” session was placed after the first method; in this session, the expert was asked questions about previous experiences of providing clinical opinion and their familiarity with statistics (characterised as “fair”, “good” or “excellent”). The purpose of the cooling off session was to: a) collect information on the level of normative expertise of the expert, and b) distract experts between the two elicitation methods, in order to limit anchoring. The disrupting effect was expected to occur as this session allowed a refresh of the short-term memory, which is likely involved in typical probability judgement tasks [24, 25].

A beta distribution was fitted in real time to the summaries provided by the experts to all four questions (one seed and one main question, for both of the elicitation methods). Experts were then asked if the fitted distribution was an accurate representation of their belief and were given the opportunity to change their summaries.

After the experts answers had been elicited through both methods, feedback was collected about the methods using closed and open questions. The closed questions addressed:

- ease of completion (*rated on a scale 1–5*, where 1 – “extremely easy to complete” and 5 – “extremely difficult to complete”);
- face validity – the extent to which the distribution reflects the expert’s beliefs (*rated on a scale 1–5, where* 1 – “not at all faithful”; 5 – “exactly as I believe”);
- which method would be preferred for a hypothetical future session.

The open questions probed about:

- any difficulties in completion or significant cognitive burdens identified during the exercise;
- any other consideration by the expert about the two elicitation methods or the entire exercise.

### Expert selection

The experts were defined to be consultant urologists or oncologists. Contact details of consultant urologists and oncologists were obtained through various networks within the research department and websites of professional associations. The experts were invited by email to participate in a face-to-face interview, which would be audio-recorded, and were given information about the project. Clinicians accepting the invitation were further contacted in order to schedule the interview.

The number of participants to include in the study was based on the available literature on expert elicitation. Cooke and Goossens [26] recommend a minimum of four experts. Other authors [27–29] suggest that the number of included opinions should be between three and five. Elicitation practitioners in environmental impact assessment recommend between six and twelve experts [30], however this number can vary a lot based on the availability of experts [6]. We agreed on a target number of eight participants.

### Elicitation sessions

The elicitation sessions were conducted face-to-face with a facilitator (BG) who walked the expert through the preparation step and through each of the questions, probing for more in-depth information, whenever possible.

The elicitation exercise and questionnaire were presented in an Excel file, with the seed and main questions presented on different sheets. The exercise was audio recorded to allow validation of contemporaneous notes taken by the facilitator.

A preparatory step was conducted to help familiarise the expert with the elicitation method. This introductory step included the following:

- background information: purpose of the exercise; expectations from the expert (elicitation of uncertainty related to the parameter of interest); confidentiality; reminder of feedback requested at the end of the exercise (on the ease of use and face validity of the methods);
- explanation of general concepts: probability, proportions, uncertainty, probability distributions;
- an example question with possible answers;
- a debiasing procedure – the expert was made aware of the main potential biases in giving their opinion and was encouraged to control them (e.g. “there are no right or wrong answers, we want to know your opinion”).

Prior to conducting the elicitation sessions, the elicitation script was piloted with colleagues from within the host institution, the Institute of Health Research, University of Exeter Medical School, to ensure the feasibility of the approach.

### Combining the individual estimates

For each elicitation method, individual distributions were aggregated across experts using the linear pooling method [31]. Expert’s probability distributions were aggregated in two ways: (1) assuming equal weights for each expert, (2) assuming weights based on calibration (see below). Calculations were undertaken in WinBUGS 1.4.3 [32], repeated for 20,000 iterations, then beta distributions were fitted to the aggregated data.

In order to achieve the most informative aggregated distribution, the relative quality of experts’ opinions was incorporated by differently weighting the individual estimates [15]. Experts were attributed weights based on how closely their answers to the seed question [31] compared to evidence available in the literature for this quantity, a process known as calibration [20].

Calibration was carried out using the “classical method” [15] modified by Bojke et al. [33]. This method considers the uncertainty in the seed data, by comparing elicited probability distributions from the experts to evidence available in the literature. In a process repeated for 10,000 iterations, the distance between the experts’ belief and the quantity in the literature is calculated. For each iteration, the expert closest to the quantity in the literature receives one point. The expert with the highest number of points is considered to be the most accurate, and their weight in the aggregation is the proportion of iterations for which they were the most accurate. This step was also conducted in WinBUGS.

While a lot of literature has been accumulated on the use of seeds [16], the use of seed-derived weights has proven difficult on clinical topics. Soares et al. [17] have explored the use of seeds and decided on using equal weighting, after observing great variability in the aggregate distributions through the use weights derived from four different seeds. In another study, Fischer et al. [18] found that by using seed derived weights, only two of 18 participating experts would be represented in the aggregate distribution. These accounts, together with the concern for the additional cognitive burden on the expert by exploring multiple seed questions at the same time as multiple elicitation methods, led to the decision of only including only one seed question in this study.

### Impact on the cost-effectiveness model

The impact of using the different aggregated distributions on the cost-effectiveness results was explored by conducting probabilistic sensitivity analysis (PSA) and associated expected value of perfect information (EVPI) analyses in the decision model. The original analysis – where the uniform distribution of uncertainty was simulated by the RAND() function – was first run, then the PSA was run four more times by replacing the RAND() function with the BETAINV() function with the corresponding parameters. The mean incremental cost-effectiveness ratios (ICER) and cost-effectiveness acceptability curves (CEAC) for analyses using each of the four aggregated distributions (weighted and unweighted histogram and hybrid methods) were compared, alongside the results of the original cost-effectiveness analysis.

## Results

### Response rate and participant characteristics

**Fig. 1:**
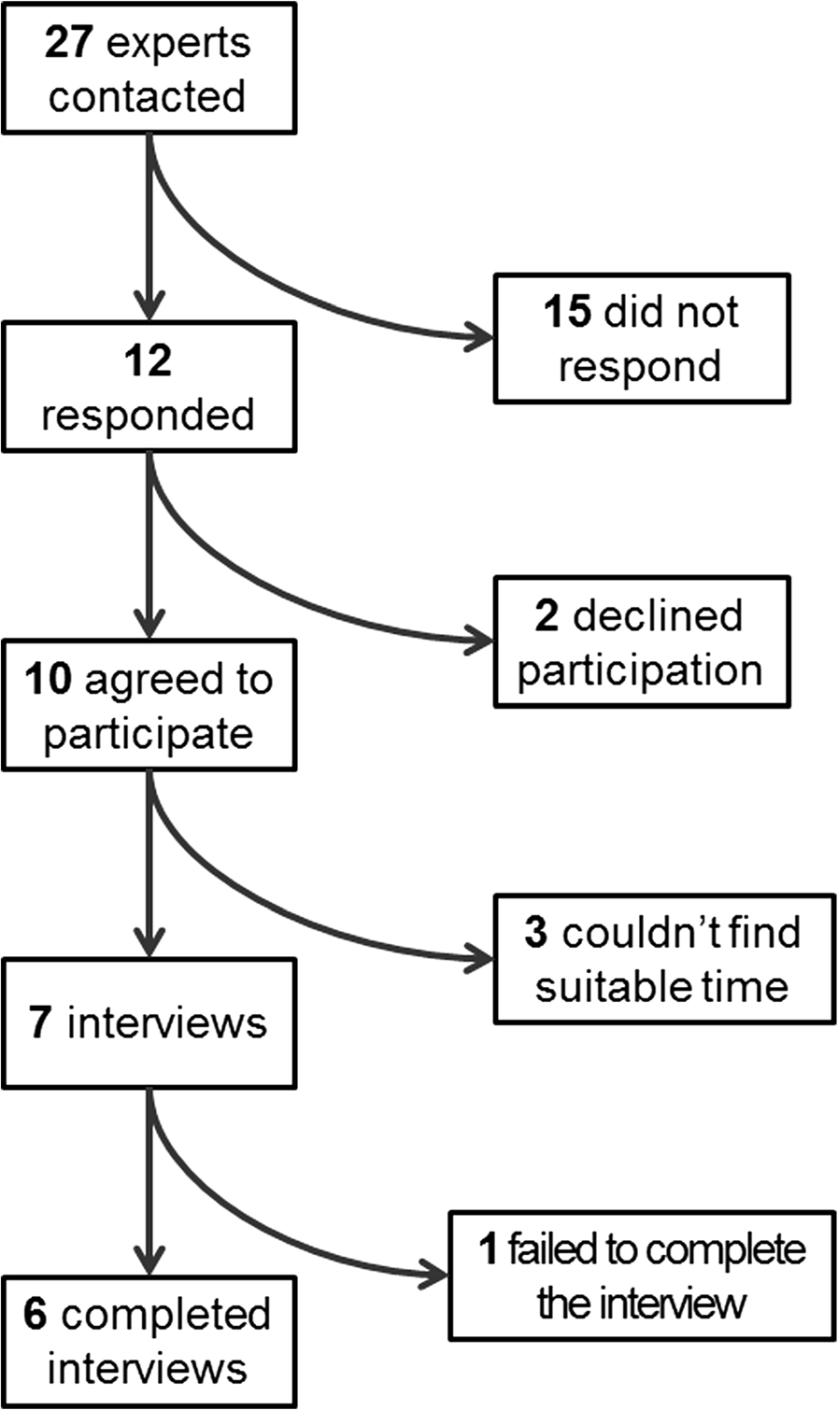
Flow diagram of experts’ participation in the study

Twenty-seven urologists and oncologists were contacted by email. Ten agreed to participate, two declined as they did not consider themselves experts in the area of interest. There was no response from the remaining 15 specialists. Of the ten experts agreeing to participate, interviews were conducted with seven, as a face-to-face meeting could not be set with the three remaining experts by the time the study was finished. See Fig. 1 for a flow diagram of experts in the study.

For the seven participating experts, the median number of days to reply to the initial invitation was 7.5, with the maximum being 23 days. Scheduling the face-to-face meeting was problematic: the median number of days to complete the face-to-face interview after the initial invitation was 98, with one case where the interview was scheduled more than six months after the initial invitation. However, when considering only the time from experts’ actually confirming their willingness to participate, it took a median of 22 days from this reply to the actual interview.

Mean interview time with experts was 28 min. For six interviews sessions, only the expert and the facilitator were present. In one session, a passive observer was also present.

One expert failed to complete the histogram method, and was therefore not included in further analysis of the elicited opinions, but was included in the analysis of the preferences.

With the exception of one expert, who claimed a “good” familiarity with statistical methods, all of the experts considered their statistical knowledge to be “fair”. All experts had some experience with providing their expert opinion, individually or in a group, but none had experience with providing quantitative judgements or specifying the uncertainty of their judgements.

### Experts’ views

Both elicitation methods were perceived to capture the experts’ beliefs equally well (median score of four), although some experts considered that the hybrid method had an advantage (E2: “you’ve got to consider things more carefully; […] it’s making me think more, might be more accurate”).

Five of the seven experts (four of the six who completed the task) preferred the hybrid method for a hypothetical future elicitation exercise. When scores from the expert who failed to complete the exercise were eliminated, the histogram method scored higher on the “ease of use” scale (median score of four compared to 3.5 for the hybrid method), but the score for the face validity of the hybrid method was slightly higher (median of 4.5, compared to four for the histogram). Scores are presented in Table 1.

**Table 1:** Distribution of scores for the seven participating experts

	Number of experts (total = 7)			
	Ease of use		Face validity	
Rating	Histogram	Hybrid	Histogram	Hybrid
1	1^a^	0	1^a^	0
2	0	1	0	0
3	1	3^a^	0	1^a^
4	4	1	5	3
5	1	2	1	3

For “ease of use”: 1 means “extremely difficult to complete” and 5 –“extremely easy to complete”; For “face validity”: 1 means “my belief is not represented faithfully at all” and 5 – “exactly as I believe”

^a^Includes the expert that did not complete the task

The expert failing to complete the exercise had difficulty understanding the histogram method and especially the relative value of the “crosses”, arguing that 20 crosses were not enough to make a histogram (E5: “It can’t be done with 20 crosses!”) and that it was difficult (when answering the seed question) to provide a single distribution, because it implied the mental aggregation of several different sub-groups of patients (E5: “It’s too broad a topic to make such a specific comment accurately”). Although a valid answer through the histogram method may eventually have been obtainable, the session had exceeded the estimated duration and the expert was becoming agitated by perceived failure to progress. Therefore the elicitation method was abandoned.

Generally, the experts found the histogram method easier to use compared to the hybrid method (median score of four for the histogram and three for the hybrid on “ease of use”). Reasons for this were the constant visual feedback (E2; “representation via a graph is easier for me”) and that it did not require precise values (E7: “I liked the idea of putting crosses on a scale”). On the other hand, the histogram method was perceived as trading face validity for ease of use (E4: “needed more crosses” [but] “I suppose more crosses would have been tedious to me”).

### Elicited distributions

Each of the six included experts provided summaries of four distributions, representing answers to the seed and main questions through both elicitation methods (see Additional file 1). Each expert agreed that the fitted smooth function was an accurate representation of their belief and that there was no need to further adjust their answers.

The audio recordings showed that experts who were very confident about the value of interest were reluctant to provide a distribution that was too wide (E3: “that’s a smudge […], a broad confidence interval on something that doesn’t happen”) and experts who were hesitant about the value (E2: “broad range, really”) indicated wider intervals.

**Fig. 2:**
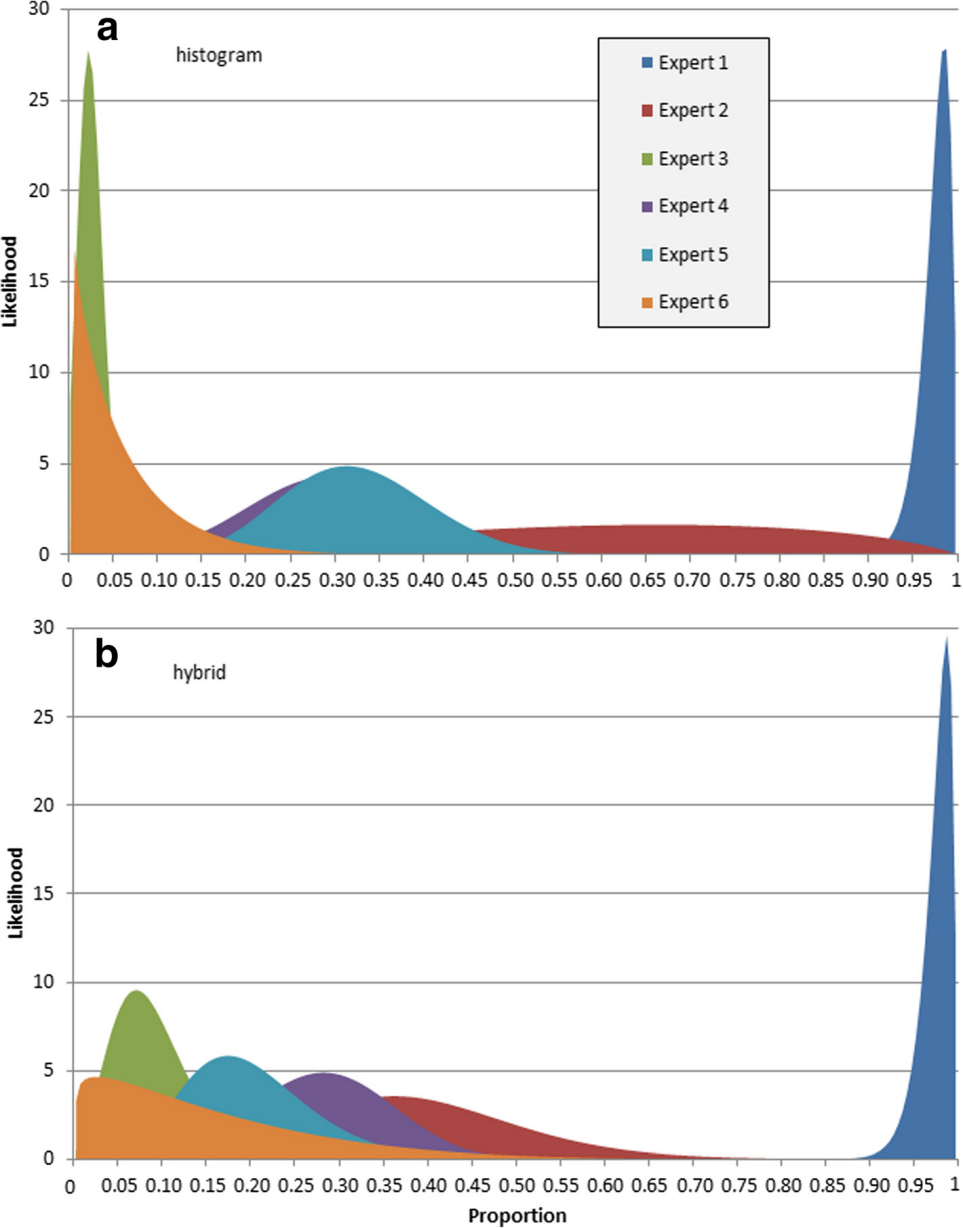
Smoothed distributions for the main question represented obtained by using **a** histogram and **b** hybrid

Individual distributions based on the elicited summaries are presented in Fig. 2. Experts 3 and 6 provided distributions with a mean closer to zero whereas Expert 1 provided a distribution with a mean close to 1. Expert 2 provided very wide distributions, while Experts 4 and 5 provided distributions similar in mean and spread.

### Calibration

**Fig. 3:**
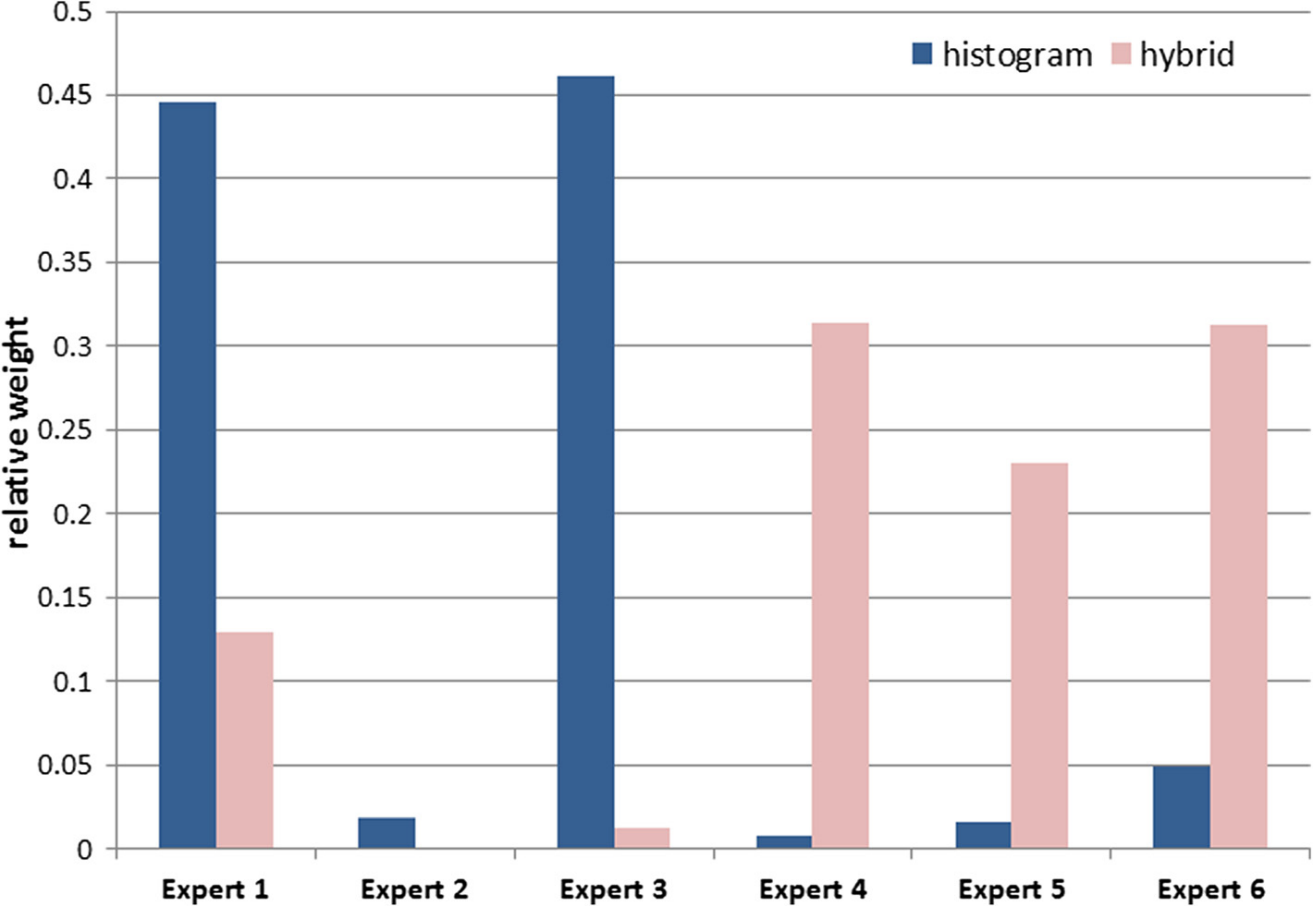
Comparison of weights attributed to the experts in the calibration step

Calculated weights for each expert are presented in Fig. 3. Based on the experts’ elicited opinions for the seed question with the histogram method, more than 90 % of the weight was given to just two experts (E1 and E3), who provided opposing responses to the main question (see Fig. 2a). With the hybrid method, weights were more evenly distributed among experts, with just one expert receiving no weight in the calibration step (E2). There were clear differences in the weighting of the experts depending on the elicitation method used (see Fig. 3).

### The aggregated distributions

**Fig. 4:**
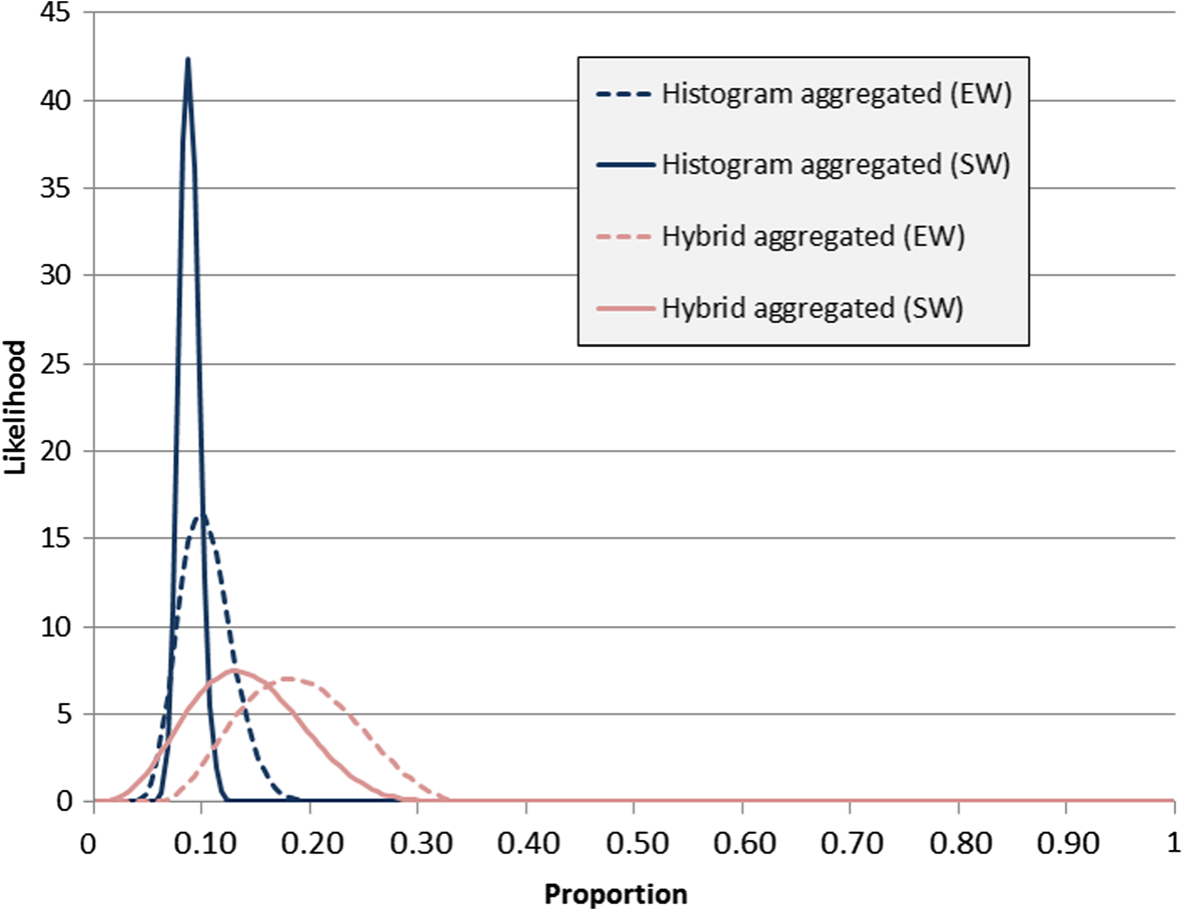
Combined distributions obtained from the histogram and hybrid method using both equal weighting (EW) and seed-based weighting (SW)

Figure 4 shows the four aggregated results – histogram and hybrid, both equally weighted and seed-weighted. The distributions from the histogram method (both seed-weighted and equally weighted) were slightly narrower than the distributions from the hybrid method, suggesting greater certainty in the aggregated distributions from the histogram method. Also of note was that the mean values from the histogram method were lower than those from the hybrid method: 0.1 vs 0.14 for the equally weighted aggregate distributions and 0.09 vs 0.19 for the seed-weighted aggregate distributions.

### Impact on the cost-effectiveness model

In the PSA, all four distributions indicated less uncertainty on the cost-effectiveness plane than in the original cost-effectiveness analysis. There was little difference between weighted and unweighted aggregate distributions from the histogram or the hybrid method.

The mean ICER was between £162,600 - £175,500 per QALY gained for the four informed distributions, compared to £134,500 per QALY for the original cost-effectiveness analysis. This did not change the model conclusions given a willingness-to-pay of £20-30,000 per QALY [34].

The impact on decision uncertainty of using the aggregated distributions reduced the EVPI value at £30,000 per QALY threshold by 74–86 % from the original analysis (see Table 2).

**Table 2:** Model results using different input values for the parameter of interest

	Mean incremental costs (£)	Mean incremental QALYs	Mean ICER from PSA	EVPI @ £30000 (£/per patient)
Original analysis	1,200	0.009	134,400	35.48
Histogram equal weights	1,400	0.008	174,200	6.35
Histogram weighted	1,400	0.008	175,500	4.93
Hybrid equal weights	1,300	0.008	162,600	9.27
Hybrid weighted	1,300	0.008	170,100	7.57

*QALY* quality-adjusted life years, *ICER* incremental cost-effectiveness ratio, *EVPI* expected value of perfect information

**Fig. 5:**
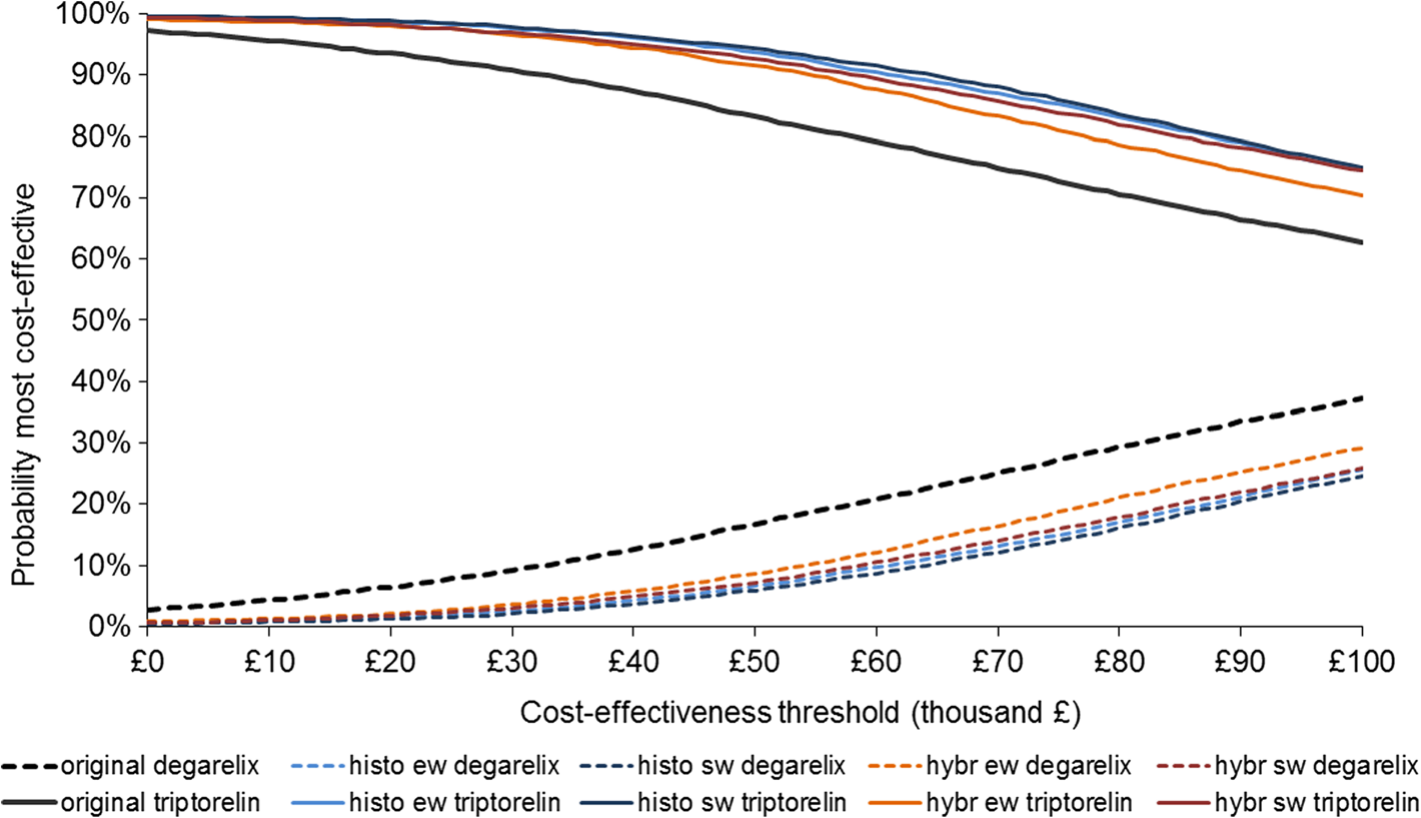
Cost-effectiveness acceptability curves using different input values for the parameter of interest. Note: ew- equal weighting of experts, sw- experts weighted on the seed

The cost-effectiveness acceptability curves corresponding to the five different analyses are presented in Fig. 5. By using any of the four elicitation-based distributions, the decision uncertainty decreases.

## Discussion

In this study differences in the methods used to elicit probability distributions from experts to populate a HTA decision analytic model have been explored.

Using the aggregated distributions based on the elicitation of expert opinion, instead of a vague distribution as used in the original cost-effectiveness analysis, our study has found a reduction in decision uncertainty. This is not surprising, given that the original model used a uniform distribution for the parameter of interest. Using an informed distribution did not result in a change in likely adoption decision, as the point estimate of the cost effectiveness of the technology is considerably more than the likely threshold for willingness to pay in the UK NHS (£20-30,000 per QALY).

Recruitment for the study proved more challenging than expected. Contacted experts exhibited little interest for participating in the study. Two possible explanations of this could be found in the literature. First, doctors tend to have a lower response rate to surveys than other categories of professionals [35]; second, clinicians are reportedly more reluctant than other professionals to give their opinion as probability judgements [36]. While more experts would have improved our ability to comment further on the findings of this study, the small sample of experts may also be a measure of the feasibility of conducting elicitation in a health technology assessment context.

We found limited differences between the elicitation (histogram vs hybrid) and aggregation (weighted vs unweighted) methods that were used, although the two elicitation methods were closely ranked by the experts in terms of ease of use and face validity.

Although many elicitation methods have been reported in the HTA literature [4], and even more in the general elicitation literature, only two elicitation methods were considered here in order to limit the cognitive burden on the experts. The same reason also contributed to the calibration step only using one seed question.

A number of measures were taken to limit the impact of anchoring (expert’s fixing on an initial value) on the elicited distributions: methods were applied in random order and a “cooling off” session was included between the first and second elicitation methods. An alternative design was considered, which would have included a longer “cooling off” period of several days, but this was seen as impractical, given the difficulty of getting enough time with the experts. On the other hand, experts were expected to have a certain degree of consistency when answering a given question (even if phrased a little differently) twice. Given this, and the limited number of experts participating in the study, the impact of anchoring is difficult to fully assess.

The generalisability of this study’s results is also limited by the number of participating experts and by the crossover design of the study.

In this study, only oncologists and urologists were included as experts, regardless of their normative expertise. It is unclear whether choosing different types of clinical experts (e.g. GPs or orthopaedists), or experts with a certain level of normative expertise, would have led to different results.

Currently, there are few comparative studies on elicitation methods in the general literature, and none concerning the use of elicitation specifically in HTA. The general view on using multiple experts is that two phenomena need to be considered: disagreement and the relative value of the individual experts [37].

An interesting issue was revealed during the calibration step with two experts, having been established as having the most weight following the calibration question, providing opposing responses to the question of interest using the histogram method. While in theory this situation is possible (with experts from different areas having varied experiences), the resulting distribution might not provide the information required to reduce decision uncertainty, negating the usefulness of a weighting step. Counterintuitive results of opinion weighting have also been encountered by other authors; for instance, in their elicitation exercise, Bojke et al. [33] report that an expert that had provided an illogical estimate for the question of interest (suggesting more disease progression with treatment than without treatment) received the most weight in the combined distribution. In addition to the challenges of selecting the appropriate seeds, some research [31, 38] suggests that simpler methods (i.e. using equally-weighted aggregation) tend to have similar performance to more complex methods (i.e. weighted aggregations) as was found in our study. More research is needed to clarify the benefits of calibration in the context of health technology assessments. We are currently preparing a paper which explores further the use of calibration in elicitation (Grigore et al., in preparation).

The incident where an expert could not complete the elicitation exercise is not unique in the literature. A number of reports were identified in a previous review where experts have had difficulty with certain elicitation methods, despite the mediation of a facilitator [4].

Mathematical algorithms exist to calibrate experts and to aggregate individual distributions, but recruiting the right expert and asking the right question is not straightforward. While a number of good elicitation practice advices are available [9, 14, 39], the decision of whose opinions to elicit (and how) is left to the analysts. A lot more could be learned about the appropriateness of different elicitation methods in future methodological studies on capturing probability distributions from experts to be used in decision models. One aspect that requires attention is exploring the benefit of methods to derive the relative weight of experts, when multiple experts are used.

## Conclusions

Expert elicitation can be effectively used to inform decision analytic models and can contribute to the decrease of decision uncertainty. Although it may prove difficult to objectively choose one elicitation method over the other, in this case the policy decision did not depend on the method used, with both elicitation methods reducing decision uncertainty.

## Abbreviations

EVPI, expected value of perfect information; HTA, health technology assessment; PSA, probabilistic sensitivity analysis; QALY, quality-adjusted life year

## Additional file

Additional file 1: Elicitation guide – the guide used in the study. (DOC 233 kb)
