# Supplementary material for: A comparison of two methods for expert elicitation in health technology assessments
Source: BMC Med Res Methodol. 2016 Jul 26;16:85. doi: 10.1186/s12874-016-0186-3 (PMC4960697; doi:10.1186/s12874-016-0186-3)
Supplement: Additional file 1: — Elicitation guide – the guide used in the study. (DOC 233 kb) [file 12874_2016_186_MOESM1_ESM.doc]

**Additional file**

**Elicitation guide used in the study**

**Introduction**

Thank you for agreeing to participate!

As mentioned in the information sheet, we would like you to give us your expert opinion on a few quantities, for which no empirical data is available. We will ask this information from you in several ways. You will be given a chance to adjust your answers and to provide feedback about the exercise.

**Background information**

We are conducting this study with two objectives.

The first objective of the study is to obtain from you information needed to inform a decision model on the cost-effectiveness of treatments for advanced hormone-dependent prostate cancer. We are not interested in a point estimate, but your belief about the entire range and the distribution of uncertainty around the value. Data collected in the form of a probability distribution allows the model to incorporate the magnitude of uncertainty, so that the model output takes into account this uncertainty.

The second objective of the study is to test several ways to elicit such information. This allows us to make a step in the direction of standardising this type of enquiry.

I will be here to guide you through the entire process and to clarify any questions that you may have.

**Informed consent, anonymity**

In order to document the process of elicitation, we will audio record this session. The records will only be used to study the process and will absolutely never be released to third parties. Any opinions or comments that we get from you will be anonymised in any publication, so that they are not associated with your name. All information that we collect will only be used to either inform the decision model or to study the application of different methods of capturing expert opinion in a probabilistic way.

If you agree to this, please sign the consent form.

**Probabilities Training**

You may find the questions a little tricky to answer. The following section is intended to make the process a little easier, by giving you an overview of what to expect and what we need from you.

Elicitation is a formal way of transforming your knowledge into a statistical form.

There are a few things about eliciting probabilities that you should be aware of. A lot of cognitive processes interfere with humans’ ability to express probabilities and introduce bias. Especially three types of bias need to be kept under control during the session. One is judgment by anchoring (fixing on a value provided earlier) – it is important that you do not refer to values provided in previous questions and try to answer each question without thinking of previous questions. Judgment by availability (attributing a higher probability to events that are familiar) is another aspect which could influence the results. Please do not limit yourself to events that easily come to mind and consider the whole spectrum of probabilities. Overconfidence (having a too narrow interval) is also a common bias - we would ask you to refrain from narrowing the interval if you are not certain enough that the value of interest is within your range.

Even if you don’t know the answer to the questions, you should still answer them; just express how unsure you are about it in the response. Remember that we are asking about data that is unknown, so there are no right or wrong answers. We are interested in your personal beliefs.

This is how your belief will be represented:

- the horizontal describes the range – the width of the range should reflect how certain you are: the less certain you are, the wider the interval;
- the vertical describes the likelihood – the height of a certain value should reflect how probable you think a value is: the more likely a value is, the taller the area above it.

**Example**

This example question will help you get an idea of what the answer could look like.

Consider all the patients that you saw this year. Now, answer this question:

What proportion of these patients own a pet?

**Example answer A:** You believe that between 30% and 55% of your patients own a pet, and it is more likely that the 45% of your patients own a pet.


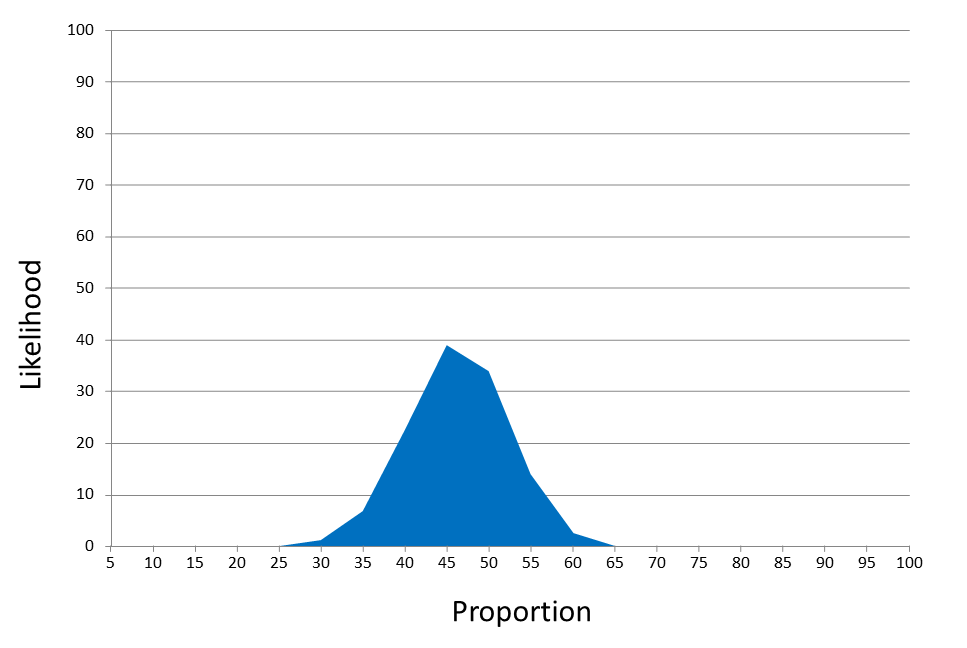


**Example answer B:** You are less certain about the true proportion, but believe that between 5% and 70%, of your patients own a pet, and somewhat more likely that 35% of your patients own a pet.


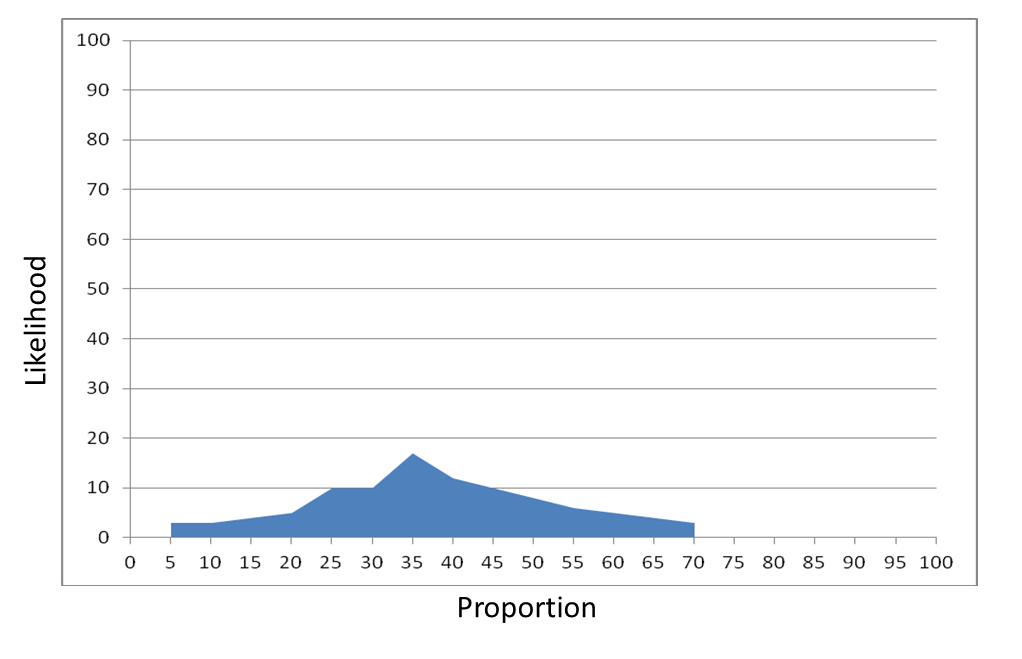


**Example answer C:** You are almost completely uncertain about the answer, although believe that it’s somewhat more likely that between 45% and 65% of your patients own a pet.


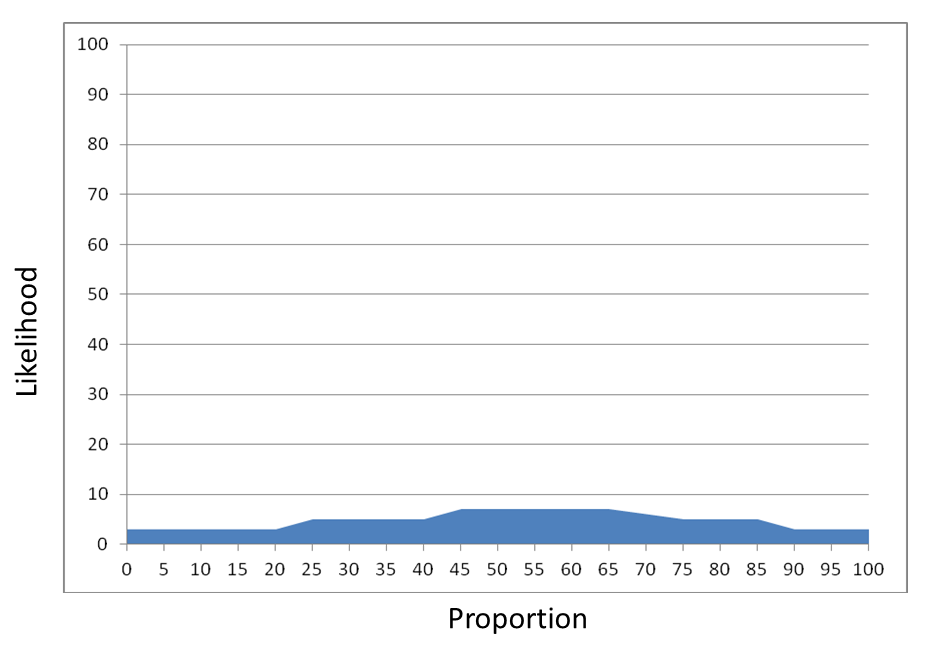


**Example answer D:** You are completely certain that 100% of your patients own a pet! Of course, this is very unlikely, but we wanted you to see an extreme case of a probability distribution.


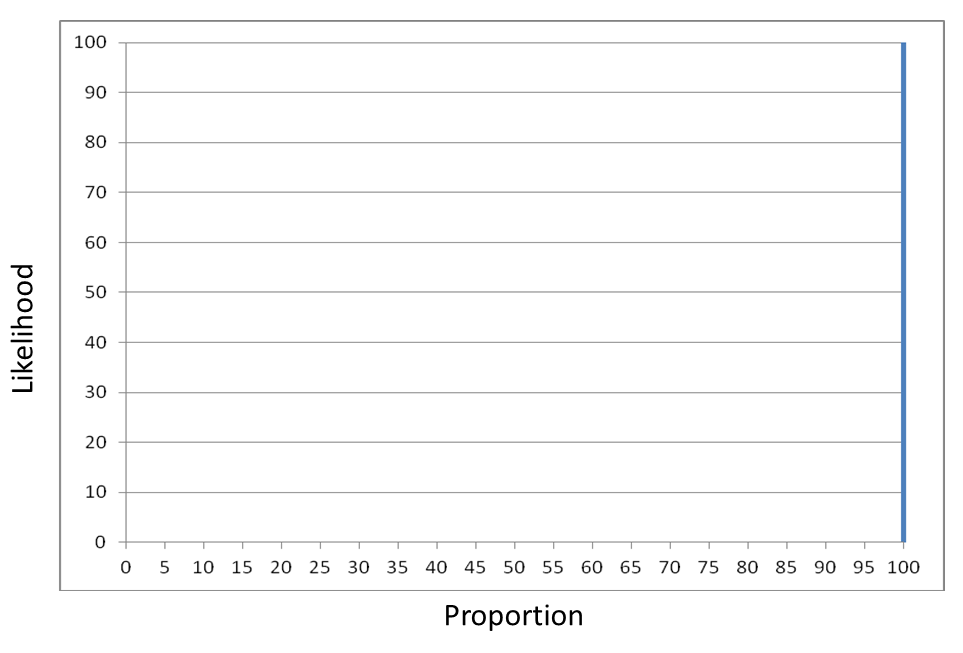


[Note: *check whether further explanation is necessary*]

Now, let’s begin with the questions!

[Note: *Methods A and B will be applied in the predetermined order*]

**METHOD A**

**Introduction:** In this exercise A, information is requested using a grid. Each column represents a range of 5% of the proportion. We request that you place 20 crosses in each grid. Each cross represents a likelihood of 5% that the true value would be in the corresponding interval. We would like you to place all 20 crosses in one or more columns to represent your current belief and uncertainty about that particular question. Start from the bottom row and stack crosses as necessary. For example, if you are completely certain about the proportion, then stack all 20 crosses in the corresponding column of the grid;

Example grid

| Frequency |  |  |  |  |  |  |  |  |  |  |  |  |  |  |  |  |  |  |  |  |  |
| --- | --- | --- | --- | --- | --- | --- | --- | --- | --- | --- | --- | --- | --- | --- | --- | --- | --- | --- | --- | --- | --- |
|  |  |  |  |  |  |  |  |  |  |  |  |  |  |  |  |  |  |  |  |  |  |
|  |  |  |  |  |  |  |  |  |  |  |  |  |  |  |  |  |  |  |  |  |  |
|  |  |  |  |  |  |  |  |  |  |  |  |  |  |  |  |  |  |  |  |  |  |
|  |  |  |  |  |  |  |  |  |  |  |  |  |  |  |  |  |  |  |  |  |  |
|  |  |  |  |  |  |  |  |  |  |  |  |  |  |  |  |  |  |  |  |  |  |
|  |  |  |  |  |  |  |  |  |  |  |  |  |  |  |  |  |  |  |  |  |  |
|  |  |  |  |  |  |  |  |  |  |  |  |  |  |  |  |  |  |  |  |  |  |
|  |  |  |  |  |  |  |  |  |  |  |  |  |  |  |  |  |  |  |  |  |  |
|  |  |  |  |  |  |  |  |  |  |  |  |  |  |  |  |  |  |  |  |  |  |
|  |  |  |  |  |  |  |  |  |  |  |  |  |  |  |  |  |  |  |  |  |  |
|  |  |  |  |  |  |  |  |  |  |  |  |  |  |  |  |  |  |  |  |  |  |
|  |  |  |  |  |  |  |  |  | X |  |  |  |  |  |  |  |  |  |  |  |  |
|  |  |  |  |  |  |  |  |  | X |  |  |  |  |  |  |  |  |  |  |  |  |
|  |  |  |  |  |  |  |  |  | X |  |  |  |  |  |  |  |  |  |  |  |  |
|  |  |  |  |  |  |  |  |  | X |  |  |  |  |  |  |  |  |  |  |  |  |
|  |  |  |  |  |  |  |  | X | X |  |  |  |  |  |  |  |  |  |  |  |  |
|  |  |  |  |  |  |  |  | X | X | X |  |  |  |  |  |  |  |  |  |  |  |
|  |  |  |  |  |  |  |  | X | X | X | X |  |  |  |  |  |  |  |  |  |  |
|  |  |  |  |  |  | X | X | X | X | X | X | X |  |  |  |  |  |  |  |  |  |
|  | 0 | 5 | 10 | 15 | 20 | 25 | 30 | 35 | 40 | 45 | 50 | 55 | 60 | 65 | 70 | 75 | 80 | 85 | 90 | 95 | 100 |
|  | Percentage | | | | | | | | | | | | | | | | | | | | |

[Note: *make sure that all 20 crosses have been placed on the grid. Check for consistency, e.g. columns are filled in from the bottom row*]

**Face validity check**

[Note: *show the histogram*] Does this correctly represent your beliefs?

[Note: *show the smooth distribution*] We extrapolated your input to create a smoother distribution. Does it still correctly represent your beliefs?

**Feedback**

[Note: ask *questions F1.A-F2.B*]

[Note: *if this was the first method, go to questions C.; else go to F3.A*]

**METHOD B**

**Introduction:** In this exercise, a distribution will be built on estimates that you provide.

Think about the value of interest and the range of values that it could take.

What would be the **lowest** possible number? [*note L*]

What would be the **highest** possible number? [*note H*]

What would be the **most** **likely** number? [*note M*]

Based on your estimates, four intervals have been calculated. Please allocate the probability that the value of interest lies within the following intervals (all intervals below must have a probability above 0% and sum up to 100%):

- [L, (L+M)/2]
- [(L+M)/2, M]
- [M, (M+H)/2]
- [(M+H)/2, H]

[Note: *check for consistency, i.e. all intervals have a probability above 0% and sum up to 100%*]

**Face validity check**

[Note: *show the histogram*] Does this correctly represent your beliefs?

[Note: *show the smooth function*] We extrapolated your input to create a smoother distribution. Does it correctly represent your beliefs?

**Feedback**

[Note: ask *questions F1.A-F2.B*]

[Note: *if this was the first method, go to questions C.; else go to F3.A*]

**COOL OFF SESSION**

**C.** I would like to ask you a few questions about your experience with statistical methods

**C1.** Have you participated before in exercises that formally capture expert opinion?

**C2.** What type of exercises? [Note: probe for: group or individual; qualitative or quantitative; specifically about elicitation of probability distributions]

**C3.** Would you consider your level of statistical knowledge to be:

| **Fair** | **Good** | **Excellent** |
| --- | --- | --- |
|  |  |  |

**C4.** We are halfway through our session. We can take a short break, if you require.

**FEEDBACK**

[Note: *questions F1.A-F2.B are asked after each method; questions F3.A-F3.B are asked at the end of the session*]

**F1.A** Please consider how difficult it was for you to complete this exercise.

On a scale from 1 to 5, where 1 means “extremely difficult to complete” and 5 means “extremely easy to complete”, how difficult did you consider this exercise?

|  | 1 | 2 | 3 | 4 | 5 |
| --- | --- | --- | --- | --- | --- |
| Exercise A |  |  |  |  |  |

**F1.B** Please think about the difficulty of this exercise. Can you indicate any specific obstacles you encountered in completing it?

|  |
| --- |

**F2.A** Now consider how faithfully this exercise has captured your belief about the elicited value?

On a scale from 1 to 5, where 1 means “not at all faithful” and 5 means “exactly as I believe”, how would you rate the face validity of this exercise?

|  | 1 | 2 | 3 | 4 | 5 |
| --- | --- | --- | --- | --- | --- |
| Exercise A |  |  |  |  |  |

**F2.B** Now please think about the “face validity” of this task (how successfully have we recorded your exact belief). Can you indicate any specific obstacles that prevented the faithful capture of your belief?

|  |
| --- |

**F3.A** Now that you have worked with both methods that we proposed, can you indicate which method you would prefer to use in a potential further exercise?

| **Exercise A** |  | **Exercise B** |
| --- | --- | --- |
|  |  |  |

**F3.B** Can you describe in a few words your choice? Is there anything that you want to add about these two methods, that hasn’t been said before?

|  |
| --- |

**F3.C** Do you have any questions about the session we had today?

|  |
| --- |

**CLOSURE**

Thank you very much for taking part in this study. Your input is very useful for our research.
